# Memory and Self–Neuroscientific Landscapes

**DOI:** 10.1155/2013/176027

**Published:** 2013-05-14

**Authors:** Hans J. Markowitsch

**Affiliations:** ^1^Physiological Psychology, University of Bielefeld, Universitaetsstraße 25, 33615 Bielefeld, Germany; ^2^Center of Excellence “Cognitive Interaction Technology” (CITEC), University of Bielefeld, 33615 Bielefeld, Germany; ^3^Hanse Institute of Advanced Science, P. O. Box 1344, 27733 Delmenhorst, Germany

## Abstract

Relations between memory and the self are framed from a number of perspectives—developmental aspects, forms of memory, interrelations between memory and the brain, and interactions between the environment and memory. The self is seen as dividable into more rudimentary and more advanced aspects. Special emphasis is laid on memory systems and within them on episodic autobiographical memory which is seen as a pure human form of memory that is dependent on a proper ontogenetic development and shaped by the social environment, including culture. Self and episodic autobiographical memory are seen as interlocked in their development and later manifestation. Aside from content-based aspects of memory, time-based aspects are seen along two lines—the division between short-term and long-term memory and anterograde—future-oriented—and retrograde—past-oriented memory. The state dependency of episodic autobiographical is stressed and implications of it—for example, with respect to the occurrence of false memories and forensic aspects—are outlined. For the brain level, structural networks for encoding, consolidation, storage, and retrieval are discussed both by referring to patient data and to data obtained in normal participants with functional brain imaging methods. It is elaborated why descriptions from patients with functional or dissociative amnesia are particularly apt to demonstrate the facets in which memory, self, and personal temporality are interwoven.

## 1. Introduction

In this review I will stress the importance of memory for a healthy self and will point to constituents that are necessary to develop and maintain an integrated interplay between information processing and self-development. Data from neurological and psychiatric patients will be used to demonstrate the challenges this interplay undergoes when somatic and psychic prerequisites are impaired or altered. Furthermore, a modern view on memory systems and processes is provided. From this data it will be concluded that there is an intimate relation between autobiographical memory and the self [1]. The data will also reveal that the self is constituted of a number of features and therefore goes beyond memory as such [2–7].

## 2. Memory Is of Survival Value and Constitutes the Essence of a Personality

Memory constitutes an essential feature of our personality. This was already stated in 1870 in a booklet by Hering [8] (which in 1895 was translated into English [9]). He wrote on page 12 (my translation):

*Memory connects innumerable single phenomena into a whole, and just as the body would be scattered like dust in countless atoms if the attraction of matter did not hold it together so consciousness—without the connecting power of memory—would fall apart in as many fragments as it contains moments.*

Hering therefore stated that human memory is necessary in our present to recruit ideas and facts from the past in order to manage our future [10]. Furthermore, memory is the glue which makes us a unique personality. However, not only we as human beings possess the ability to store information perceived in the environment. Every animal learns about its social and biological environment. Long-term storage of information has most likely a survival value: For an animal it is helpful to maintain information on good and bad food, as healthy and tasting food prolongs life and is emotionally satisfying. On the other hand, the avoidance of poisonous food helps to maintain a healthy body and to avoid being intoxicated. In this way, memory has survival value for an individual. Similarly, it can be proposed that memorizing odors of members of the own and of other species allows to infer whether another individual is ready for mating or may be aggressive. We all know that not only dogs communicate to a considerable extent via sniffing, but that also many rodents, carnivores, and ungulates mark their territory so that another male is likely to avoid their territory and thereby avoids serious injuries from being attacked by a stronger individual. In this way survival of the species is prolonged via memory.

These examples also point to brain regions which early in phylogeny were mainly engaged in odor [11] but later on in phylogeny were necessary for processing emotions and memory. Even in human beings it has been found that our first memories are strongly odor related: Willander and Larsson [12] presented odor, word, and picture cues to elicit past memories from adults and found that the odor cues elicited mainly memories from the first decade of life, while the word and picture elicited mainly past events from the second decade of life. The brain regions, initially (in phylogeny) relevant for odor processing, are subsumed under the heading “limbic system” [13] and are discussed as mediating between the phylogenetically old structures of the brain stem and the advanced structures of the neocortex [14–16]. Among them, the amygdala is necessary for a proper interpretation of the social and biological environment [17–22] and the hippocampal formation for the transfer of memory from short-term to long-term storage and possibly also for retrieval [23]. Other limbic and paralimbic [24, 25] structures such as the septal nuclei [26], medial and anterior thalamic nuclei [27] support the emotional colorization of memories and are nowadays seen as overlapping with the so-called default mode network [28, 29], a system of structures active when the brain is at wakeful rest and not focused towards stimuli in the outer world. Recent data indicate that this system provides the neurophysiological basis for self-processing operations such as first-person perspective taking and an experience of agency [30, 31].

## 3. The Development of Memory and the Self

It is obvious that human babies have at best tiny spoors of memory from their prenatal life. They first start by exploring the environment with their senses—from odor to the haptic sense. By doing so they learn to associate environmental objects with specific feelings—that the wooden frame of the bed may be resistant and harmful when touched too vehement, or that the breasts of the mother smell and taste well. Thereafter they continue by associating objects with heard expressions and gradually acquire language. And even later they start to understand the meaning of time [32, 33]. Theory of mind functions follow both with respect to cognitive and affective situations (becoming able to make inferences on another person's intentions and becoming able to make inferences on how he or she might feel) [34–36]. Only when having reached this state of mind, a self becomes established and autobiographical memories are possible [37–40]. However, even then there are of course concepts of the self that are only established later during adolescence [41–45].

The three main constituents for the development of autobiographical memory and the self are the development of language and the maturation of the brain [46, 47]. Of course, this process is culture shaped as well [48, 49] and continues into later childhood [50, 51]. Interestingly, adults are not aware of their first years of life; we speak of infantile amnesia. Memories of about the first three years of life are not consciously remembered at adulthood [52–55]. As Harpaz-Rotem and Hirst [53] showed, the birth order has an effect: first-born children or single children develop autobiographic memory and a self earlier than children appearing later in birth order (i.e., later-born children have their first memories stemming from a later age as adults). This is most likely due to the fact that the first-born child is addressed individually by parents and peer group, while later born children are addressed as a group. This hypothesis is confirmed by the fact that in societies where children are treated less individually as in a kibbutz [53] or in East Asia [54–56], they also develop autobiographical memories and a self later in time. For a more detailed discussion of this topic see the review of Markowitsch and Staniloiu [57].

Also the brain representation of the self may differ between Westerners and East Asians. Zhu et al. [58] found with functional brain imaging that Westerners used the medial prefrontal cortex exclusively for representation of the self, while Chinese people used it for representation of the self and of their mother. (It should, however, be mentioned that these data hold for individuals with normal brains. As Philippi et al. [59] stressed, the situation in patients with damage to the medial prefrontal cortex speaks for functional reorganization and for the use of more distributed neural networks for representing the self.)

## 4. What Constitutes the Self?

Everyone has an implicit idea on what the self is. That we are ourselves is evident if we are adult, healthy individuals. The situation becomes different when we ask whether a human baby has a self, a patient with severe Alzheimer's disease has a self, or a water snail has a self. Apparently “selves” are unequal. Simple animal species have in common with human beings that they possess a body (“embodiment”—subjective experience of a body [60, 61]), they have a metabolism and a nervous system. Most species can avoid too much light or heat or coldness; also human babies try to avoid pain and to get food. Other species are not only aware of the fact that they want to live in a kind of homoeostasis, but they are able to cheat and to communicate. And still others may be able to recognize themselves in the mirror, including apes, whales, elephants, and human children of about 18 months of age [62–64]. Butler and coworkers [65] recently raised the question whether there are differences in recognizing oneself in mirrors, or on photographs, or in TV, and so forth. Other scientists alerted to the fact that there might be a difference between self-recognition ability in species living in groups or hordes (e.g., gorillas) and living isolated as individuals (e.g., orangutans). Self-recognition is nevertheless recognized as a distinct feature of self-awareness or self-consciousness. However, everyone knows that self-awareness does not make apes equal to human beings—apes do not build skyscrapers and do not know that they will die. Several researchers—such as Damasio [66]—argue that there are hierarchies of consciousness. For Damasio there is the very simple “protoself” (important for homoeostasis), thereafter comes the “core self”, and then the “extended self”. The core self resembles core consciousness that allows animals to be aware and to react to their environment. The “extended self” requires a “personhood”, a self-identity, and consequently autobiographical memory. Though Damasio argued in part differently by attributing an extended self also to some animals, Tulving [67] proposes that only human beings should possess this, as only human beings “possess “autonoetic” episodic memory and the ability to mentally travel into the past and into the future, and that in that sense they are unique” (page 4). One might add that even not all human beings possess this feature—small children, severely brain-damaged, or mentally retarded patients are not able to mentally travel into past and future (see [57] for a more detailed discussion of this topic).

The self, as it is used in this paper, is seen as based on autobiographical memory and on “autonoetic consciousness” or “autonoesis” in the sense of Tulving [67]—being aware of one's being in time, both respect to one's past and one's “proscopic chronesthesia” (looking into the future, imaging the future). (In 2002 Tulving [68] described “autonoetic” as the “special kind of consciousness that allows us to be aware of subjective time in which events happened”; page 2.) Tulving [68] used the example of an Estonian children story to introduce the “spoon test”: A child of probably more than four years of age “dreams about going to a friend's birthday party where the guests are served delicious chocolate pudding, her favorite. Alas, all she can do is to watch other children eat it, because everybody has to have her own spoon, and she did not bring one. So the next evening, determined not to have the same disappointing experience again, she goes to bed clutching a spoon in her hand.” (page 44). This test requires looking back into the past and looking forward into the future, without being triggered by an actual environmental event [69–71]. Having described the spoon test, this leads to the question of what constitutes (a) memory.

## 5. What Is Memory?

Similarly as with the term self that of memory is universally understood, but nevertheless not easy to define. Already some decades ago Sinz [72] on page 19 of his booklet gave the definition in which he wrote (my translation):

*With memory we mean the learning-dependent storage of ontogenetically acquired information which is incorporated into neuronal structures in a selective, species-dependent way, and which can be recalled at arbitrary time points, that is, which can be made available for a situation-dependent behavior.*

This definition emphasizes that the information is not genetically available but acquired postnatally via learning, and that different species may have different neural structures for incorporating and storing information. All this is of importance as it is known for instance that species have a genetically programmed repertoire of behaviors which is not learned (for instance, nest building in birds or the sucking, grasping, and swimming reflexes in human babies). Furthermore, species with a short life time naturally must have shorter consolidation periods for newly learned information than species with a long life time. 

Recently, we have, however, accumulating evidence for major interplays between genetics and learning: the new field of “epigenetics” [73–78] refers to heritable changes in gene expression which do not change the DNA sequence, but still may be transmittable transgenerationally [79, 80]. Epigenetics describes mechanisms by which experiences (environmental stimulation) can modify gene function and gene expression [81]. With respect to learning and memory, epigenetic modifications become of major importance [82–88]. Such results have broadened the definition of memory. Rensing et al. [89] proposed that the term “memory” might be used for plants and viruses as well and should include the following features:

- selection (and/or modification) of preexisting information,
- activation of specific (selected) networks by information to be memorized,
- consolidation of the memory networks in a latent state (standby mode),
- retrieval of memory networks.

“Selection”, “activation”, “consolidation”, “stand by,” and “retrieval” (or “reactivation”) are the important terms in this model, which, however, characterize more traditional ways of memory processing as well, namely, “encoding” of information, “consolidating” it, “storing” it, and “retrieving” it. It should be emphasized here that retrieval of information leads to reconsolidation in the present state of circumstances, it in the present internal state of the individual (mood), and in the state of the presently acting environmental stimulation. Because of this, memories can act altered with repeated reactivation which may lead to the so-called false memory syndrome [90–93]. Tulving [67, 68] and, before him, Semon [94] have emphasized the state dependency of memory, implying that encoding and retrieval of information are modulated by the respective states of individual and environment. This means that if a person is in a depressive state, he or she will more likely encode the new information in a negative manner or will recall old information by emphasizing its negative aspects. State dependency also implies that it is ideal for retrieval, if the retrieval mode (condition) equals the encoding mode (condition) [95, 96].

### 5.1. False Memory Syndrome

**Figure 1:**
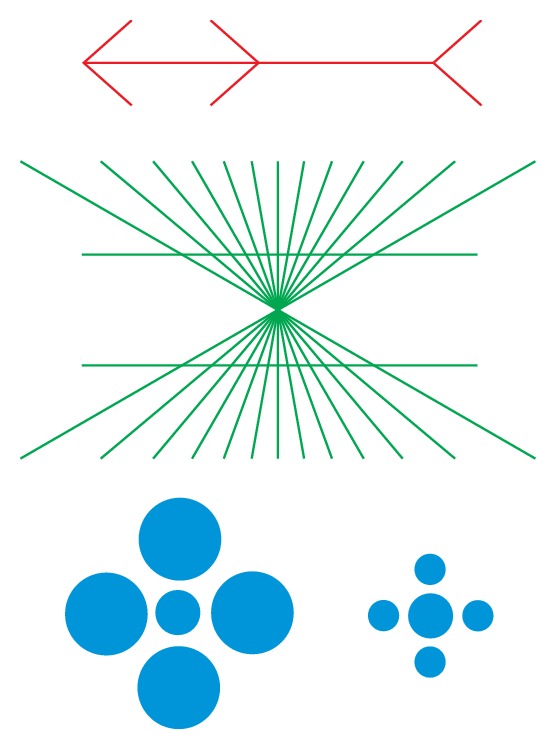
Examples of perceptual illusions.

The observation that information may be retrieved differently from how it was encoded has to be traced back to the process of perception. We know from numerous perceptual illusions (Figure 1) that our sensory systems interpret information subjectively and not physically correct. This implies that portions of the perceived information are inadequately encoded and stored and will consequently also be inadequately retrieved. Furthermore, the encoding specificity principle [97] which implies that memory is best when the same information present at encoding is also present at retrieval may lead to various forms of memory distortions if violated. Vice versa, Weingartner et al. [98] long ago have verified what had been known anecdotally before, namely, that encoding under alcohol intoxication should be followed by retrieval under alcohol intoxication. If individuals are sober at retrieval while they had learned the words while intoxicated, they performed poorer than in the matched condition. 

**Figure 2:**
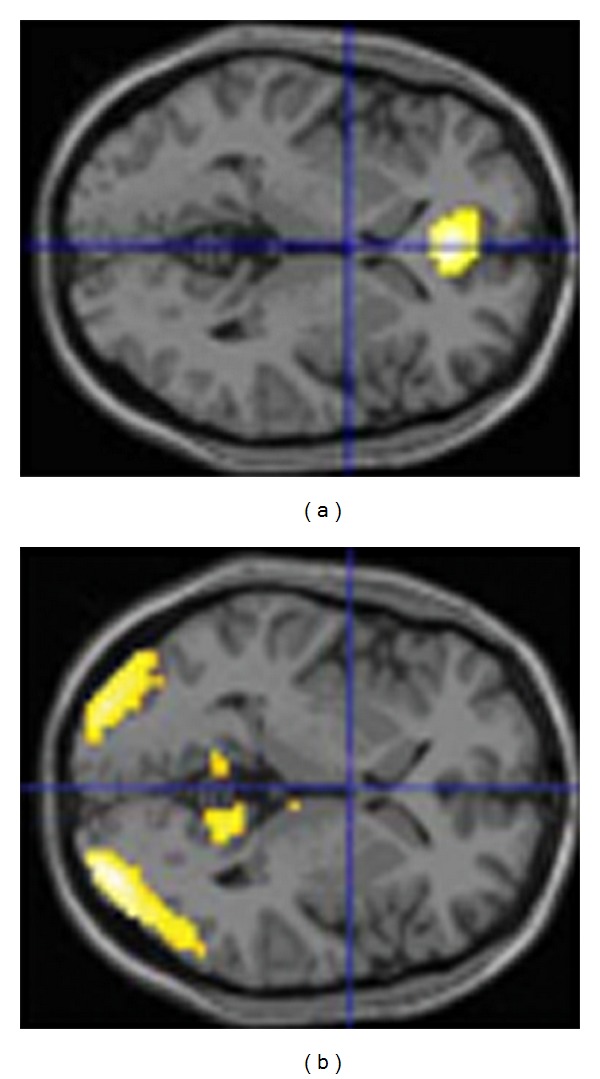
Horizontal sections through a human brain, illustrating activations (yellow) when providing correct responses to two movies (a), or when providing incorrect answers (b). Incorrect responses resulted in activations in the visual association cortex and the precuneus which can be interpreted as reflecting mental imagination and matching processes. Activations towards correct responses were found in the medial prefrontal cortex and may reflect processes of sureness.

We have investigated the phenomenon of false memories (which had been a major topic of Freud [99–101]) with functional brain imaging [102]. University students viewed two short movies (less than 8 minutes altogether) and thereafter were positioned in a magnetic resonance imaging scanner where they saw single shots from the movies, related pictures which, however, had not been seen in the movies, and unrelated pictures. Their task was to decide whether they had seen the pictures or not. Surprisingly, they gave nearly 45% wrong answers by stating that they saw a picture that had not been part of the movie or that they did not see a picture which in fact had been part of the movie. Even more astonishingly was that the brain activations towards false versus correct responses revealed a distinct pattern with true responses showing medial prefrontal and false responses posterior activations in the visual association cortex and the precuneus, all bilaterally (Figure 2). While this might be interpreted as showing that the “brain's responses” were more distinct and predictable than the behavioral responses, some caution is advisable. Subjects probably would have liked to respond more frequently with “I do not know” or “I'm not sure”; however, they were forced to press a “yes” or a “no” button. Consequently, the activations may reflect a somewhat artificial clarity or distinctiveness.

### 5.2. Possible Implications for Jurisdiction

Nevertheless, these results—together with related ones [103–109]—indicate that it is in principle possible to obtain neural correlates for false responses. It also might be possible in the foreseeable future to detect with functional brain imaging whether a patient is simulating pain or feels it indeed [110]. Such findings may have similar implications for court decisions as those on lie detection [111–114].

After questionable results with measures of the galvanic skin response [115, 116] or with thermal imaging (flushing) [117], researchers more recently have started to use functional brain imaging in order to differentiate lie from truth. This research probably started with a functional positron emission tomographic study [118] which was based on two similar ones where the neural correlates of true autobiographic retrieval were investigated [119, 120]. We found that true memories led to activations in the right amygdala and right temporofrontal region, while lies resulted in—somewhat similar to the results of our false memory study [102]—activations in a region around the precuneus. Later studies, performed with functional magnetic resonance imaging, provided partly inconsistent results [121–140], though, as Spence [132] stated, some consistencies. Among them were longer response times during lying compared to telling the truth, greater ventrolateral and anterior cingulate activity during lying, and no brain regions where truth elicited greater activity than lies. Of course, this kind of research, which already led to practical applications in the court, has been criticized as being unethical [141].

Numerous other techniques, based, for example, on electrophysiological recordings [142] or smart questioning [143], have been proposed and there is a wide variety of so-called symptom validity tests available which also have been named lie detection tests. Most of these test memory in a way that patients assume it is a difficult test, while in fact it is rather simple. Some are based on probability estimates. Examples are the test of memory malingering (TOMM) [144–146], the word memory test [146–148], and the Amsterdam short term memory test [149, 150]. M. Martins and I. P. Martins [151] evaluated malingering criteria of the word memory test and Bolan et al. recently compared advantages and disadvantages of these three tests [152]. A problem of these tests lies in the fact that if patients show a lack of effort, they perform as poorly as if they would consciously lie [153, 154]. Nevertheless, in clinical assessments, especially when patients demand compensation, these tests are used regularly. Interestingly, Sip et al. [155] recently found with functional brain imaging increased activity in the left temporal pole and the right hippocampal and parahippocampal regions when participants believed their false claims could be detected, but not when they thought the lie detector was switched off.

### 5.3. Memory and Time

**Figure 3:**
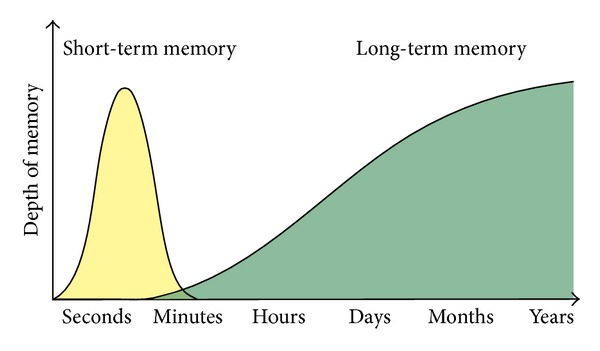
Sketch of the distinction between short-term and long-term memory and relations between memory strength and duration. It is commonly assumed that short-term memory lasts seconds to a few minutes (digit span, 5 bits of information), while long-term memory may last lifelong. Evidence for the division into these two memory systems comes from experimental psychological research and from findings in amnesic patients. Word list learning (of about 10–20 words) reveals the so-called primacy and recency effects: the first and the last perceived words are remembered best and those in the middle worst. It is assumed that the words perceived first have already been transmitted for long-term storage (recency effect), while those perceived last are still in the short-term memory storage (primacy effect). Amnesic patients usually have a preserved short-term memory ability and would show a primacy, but no recency effect.

As mentioned above, recalling mnestic information requires that it has been stored successfully before and therefore also successfully encoded and consolidated. Memory is therefore embedded in time. This is above all reflected in the usual distinction between short-term and long-term memory with short-term memory lasting seconds to a few minutes and long-term lasting for longer time periods, for some information lifelong [23].  Figure 3 depicts this time-based processing of information and Table 1 provides definition for terms which are relevant to psychological memory processes.

**Figure 4:**
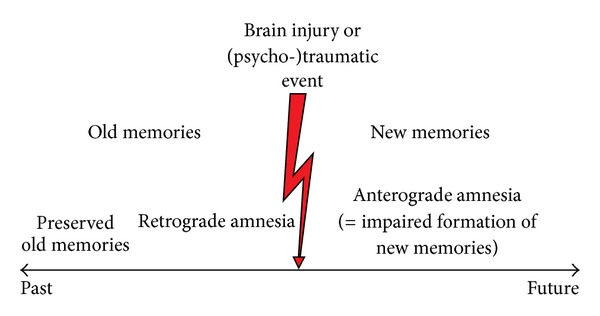
Possible consequences of brain injury on old and new memories. Anterograde amnesia refers to the inability to store new information (usually new biographical events) in long-term, while retrograde amnesia refers to an inability to retrieve old, already stored memories. Retrograde amnesia is usually unequally distributed in that way that the information closer to the present or closer to the significant event represented by the flash symbol is more easily lost than information from the remote past. This distribution was first described by Ribot [156] and is named after him (“Ribot's law”) or termed the “law of regression.” It was also Ribot who attributed three meanings to memory: “the conservation of certain conditions, their reproduction, and their localization in the past” (page 10).

**Figure 5:**
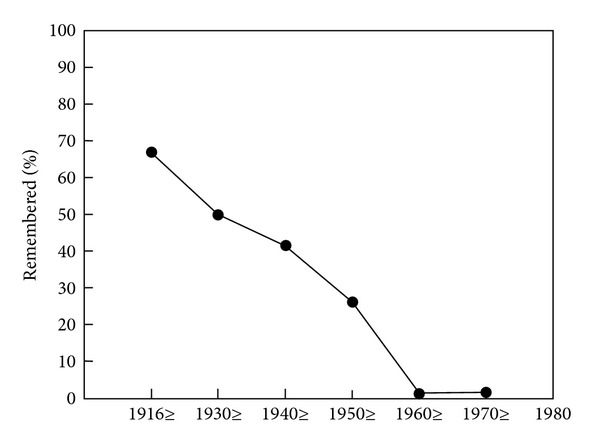
Percentage of remembered autobiographical events across the life of a patient with Korsakoff's amnesia who had written his biography prior to becoming ill [157]. The curve represents an example for the validity of Ribot's law.

Alternatives to the short-term long-term memory dichotomy such as a third, intermediate memory system [158] or the depth of encoding idea of continuous memory processing [159] did not withstand times and counterevidence. However, there is another time-based distinction which is of major importance, namely, that between anterograde and retrograde amnesia (Figure 4). After a brain infarct or after traumatic brain injury, or after a major psychotraumatic or stressful event, memory can be disturbed in two ways or in mainly one of two ways: The ability to store new information in long-term may be impaired, or the ability to retrieve old information which already had been stored in the brain. Retrograde amnesia frequently follows a time gradient so that very old information is largely preserved, while information from the time close to the injury or the stressful event may be blocked or unavailable for conscious retrieval. The preservation of old information is explainable by the facts that it came into a fresh, “empty” brain, that it probably was the first of its kind (i.e., it had the character of uniqueness), and that it was probably retrieved numerous times since its initial storage and therefore also reencoded and reconsolidated repeatedly. This relationship was already known to the French medical doctor Ribot [156] and is depicted in Figure 5. This figure stems from testing the retrograde memory of a retired psychology professor who had developed Korsakoff's syndrome [157], a disease leading to degeneration of the medial thalamus and the mammillary bodies and to massive anterograde and variably intense retrograde amnesia.

### 5.4. Memory and Contents

**Figure 6:**
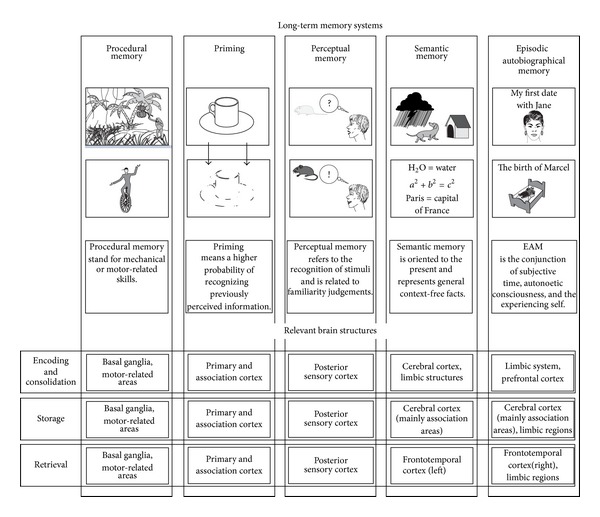
The five long-term memory systems and their assumed brain bases. Procedural memory is largely motor based but includes also sensory and cognitive skills (“routines”). Priming refers to a higher likeliness of reidentifying previously perceived stimuli. Perceptual memory allows distinguishing an object, item, or person on the basis of distinct features. Semantic memory is context-free and refers to general facts; it encompasses general knowledge of the world. The episodic autobiographical memory (EAM) system is context specific with respect to time and place. It allows mental time travel. Examples are events such as the last vacation or the dinner of the previous night. The terms “remember” and “know” describe the distinction between EAM and semantic memory, as remembering requires conscious recollection embedded in time and space and with an emotional flavoring, while knowing represents a simple, though conscious, yes/no distinction without further connotations. Tulving [67, 68] assumes that during ontogeny (as well as during phylogeny) memory development starts with procedural memory and ends with episodic autobiographical memory, a system that he reserves for human beings, while all other systems can be found in animal species as well.

The memory impairment in most patients with brain disease or a psychic blockade of memory retrieval (named “mnestic block syndrome” [164–169]) largely is confined to one system that we name the episodic autobiographical memory system [23, 57]. From the fact that this memory system is particularly vulnerable to adverse environmental or brain conditions it follows that there are other memory system. Aside from short-term memory which implies the online holding of limited bits of information for a very limited time (cf. Figure 2)—and which was extended by Baddeley [170–172] to “working memory” to reflect the fact that we also during retrieval partition information in manageable bits—we distinguish five long-term memory systems as depicted in Figure 6. These systems originate from a more limited number, namely, into two basic ones, one for largely automatically motor routines or procedures or habit, and another one for consciously processed facts and events. These memory systems are considered to evolve phylo- and ontogenetically in the same sequence starting with two principally subconsciously acting ones, “procedural memory” and the “priming system,” and continuing with “perceptual memory,” “semantic memory,” and the “episodic autobiographical memory” system, all of which are principally processed in a conscious, reflected manner (cf. Figure 6).

“Procedural memory” is used when handling a bike, driving a car, playing piano or chess, and so forth. We are not aware of the individual motor acts during performance—they are routine. For instance, when asking someone “what do you have to do first, when you want to change from the second into the third gear?”, one frequently obtains the answer “to press the clutch.” While in fact one first has to release the right foot from the gas pedal. On the same level as “procedural memory” “priming” acts. “Priming” refers to unconsciously perceived stimuli which nevertheless are incorporated in our nervous system and may, if later perceived again in the same (“perceptual priming”) or in a similar way (“conceptual priming”), lead to an easier or more frequent orientation to them, or to easier retrieval. In fact, psychologists have found that utmost of what we perceive we process unconsciously. With “perceptual memory” we reach the level of conscious information processing. “Perceptual memory” stands for familiarity processing, knowing what an item or object is, even at a presemantic (or nonsemantic) memory level; for example, to identify an apple independently of whether it is red or green, intact or half-eaten and also to be able to distinguish it from a peach or pear. “Semantic memory” deals with context-free facts, our school, and world knowledge. Finally then we speak of “episodic autobiographical memory” when personal events or episodes are meant. These refer to the conjunction of autonoetic consciousness (so the ability to reflect about oneself), subjective time perception, and the experiencing self. That is, we combine our ability to subjectively travel in time back and forth with the consciousness of our internal and external states of being. Tulving introduced his SPI model (SPI = serial, parallel, and independent) in 1995 [173], for which he stated that information is encoded into systems serially, stored in different systems in parallel, and can be retrieved independently. Hodges [174] and others proposed that processing of individual-centered episodes or events which are traceable with respect to time and place will, when repeated, finally lead to generalizations which are known as semantic or procedural memories. The so-called process of semantization [cf. Figure 2 in [57]; [175]] is in opposition to the fact that ontogenetic learning starts in childhood with semantic facts and only later includes personal events [37, 38].

Another classification of memory system divides between “declarative” and “nondeclarative” memories [176, 177]. Declarative memories are in Squire's [176] definition both semantic facts and episodic (autobiographical) events. This combination is, however, less fruitful for science and for the distinction of impaired and preserved functions in brain-damaged individuals, as usually semantic facts processing is largely preserved after brain damage, while that of episodic autobiographical events is largely impaired.

## 6. Memory and the Brain

**Figure 7:**
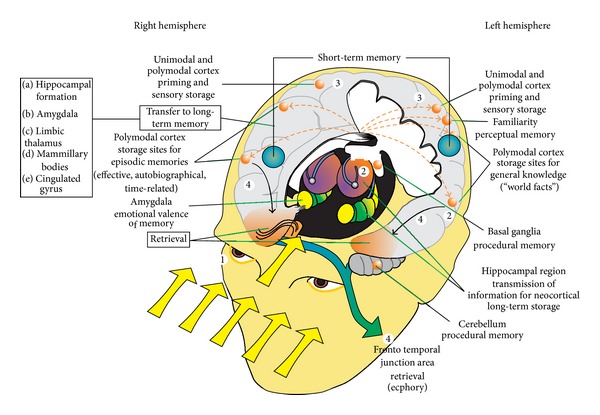
Sketch, illustrating in a condensed fashion relations between information encoding, storage and retrieval, the memory systems, and principally engaged brain regions. It is assumed that information enters the (inner) brain via the sensory organs and is then processed further, depending on the attention that is devoted to its contents. Most of the information is processed implicitly and therefore unconsciously—primarily via unimodal cortical areas. Nevertheless, much of this information remains available for subsequent analysis or even retrieval (in the appropriate—that is, triggering—context). That information which is, however, relevant for our conscious life—predominantly the semantic and episodic autobiographical information—is processed further via a number of regions and regional networks. Among these, the short-term or online holding of information is done via regions in the dorsolateral prefrontal and—at least for human beings—in the left lateral parietal cortex. From these regions semantic and EAM information is loaded into regions of the limbic system, where two circuits, the medial and the (baso-) lateral limbic circuit, are necessary (and for most instances) essential for a further analysis and for processes of associating, binding, and comparing with already existing memories (Figure 8). The limbic system with its two main circuits is supposed to be necessary for encoding and consolidating memories and for transferring them for long-term storage. Storage of most memories is proposed to occur largely within major areas of the cerebral cortex but assumed to extend beyond these, especially for EAM forms of memory. A hemisphere-specific preponderance is assumed to exist in that way that EAM information is related to the right cerebral cortex and information from the knowledge system to the left. Retrieval (ecphory) of semantic and EAM information is assumed to be triggered by structures in the temporo-frontal cortex [160–162], again with a hemispheric asymmetry. EAM, which is emotionally colored, relies predominantly on the right, while semantic memory is mainly activated via the left hemisphere [23, 163] (which also contains the Broca and Wernicke speech areas). For procedural memory the basal ganglia and portions of the cerebellum are implicated in all stages of processing from encoding to retrieval.

From ancient time onwards there were diverse ideas that intellectual functions have a representation in the brain [177, 178]. Especially the ideas of phrenology became quickly popular all over the world in the 19th century [179]. Later on there was a debate about localization or antilocalization in the brain [180] which continues until today [181]. Indeed it is likely that there is a combination of specialized and distributed processing in the brain [182–187]. This is depicted for various forms of memory processing in Figure 7. It is assumed at present that there are specialized regions for sensory and perceptual processing, encoding (short-term memory) and consolidation (limbic system; [11, 13]), storage (widespread cortical nets), and retrieval (temporofrontal cortical regions) of information.

Bilateral brain damage to structures of the limbic system [13, 188] leads to massive impairments in anterograde memory processing, both on the semantic and episodic autobiographical level [189–191]. This was found long ago with the advent of the Korsakoff's syndrome [192–199] and continued with the finding that hippocampal damage leads to amnesia [200] which, however, as published in German language, was not recognized internationally and therefore resulted in bilateral resection of this and related medial temporal lobe structures by Scoville and Milner [201] with the consequence of lifelong anterograde amnesia [189, 202, 203]. At present, much research performed both on patients with hippocampal damage (as, e.g., in neurodevelopmental amnesia [204–208], but also in patients with other etiologies [209–213]) and in normal subjects with functional brain imaging [23, 214–225] tries to reveal the specific role of the hippocampal formation (or of portions of it) in long-term memory processing. Recently, it was proposed that the hippocampal formation might also be engaged in some forms of nonconscious memory [226–229], in working memory [230, 231] and spatial memory [232–236]. Furthermore there is evidence that exercise enlarges hippocampal volume and improves memory performance [237–239] and that the hippocampal formation is engaged in sleep-related memory consolidation [240, 241].

**Figure 8:**
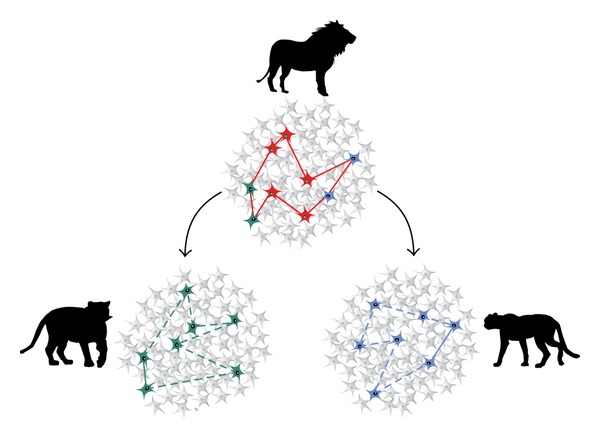
Three hypothetical neuronal assemblies, the top represents the concept of a lion, the bottom left one that of tiger, and the bottom right one that of a cheetah. The neurons interconnected by lines within each assembly represent the concept of the respective animal. The two neurons marked in green in the top assembly overlap with the circuit representing the concept of a tiger and the two neurons marked in blue in the top assembly overlap with the circuit representative for the concept of a cheetah. Quian Quiroga [182] assumes that partly overlapping representations might be the basis for learning of associations and conscious memories. (After Figure 5 of Quian Quiroga [182].)

Coming back to the question from above on localization versus antilocalization of brain functions [242], Quian Quiroga [182] promoted the idea of a compromise which is, however, inclined towards the direction of a close localization of function. Based on earlier research in the medial temporal lobe [243–246] he proposed that there are concept cells in the medial temporal, particularly the hippocampal formation, neurons that respond in a selective and abstract manner towards persons (faces) or objects. Quian Quiroga [182] proposed that small assemblies of such neurons fire distinctly to the representation of a person or an object. Furthermore he argued similarly to the idea of previous researchers [184, 186, 187] that individual neurons of such an assembly may be engaged in the representation of similar (i.e., easily associable) objects or persons. In this way they do not represent “grandmother cells” in the traditional way proposed about 50 years ago but are still rather distinct agglomerates of neurons representing a single item.  Figure 8 demonstrates how these assemblies are distinct but nevertheless overlapping with respect to related representations (such as tiger, lion, and cheetah).

### 6.1. Information Consolidation and the Limbic System

Since the end of the 20th century, scientists proposed a special role of limbic system structures in information encoding and consolidating for long-term storage [247]. These ideas originated from a number of single case reports of patients with bilateral damage to various structures of the limbic system, especially of the diencephalon [200, 248–258]. In 1937, Papez [259] published an influential paper on “A proposed mechanism of emotion” in which he suggested that interconnections between several limbic structures are necessary for a proper processing of emotions. His suggestion was later taken up and this circuit or modifications of it were proposed as essential for memory transfer for long-term storage [260, 261]. 

**Figure 9:**
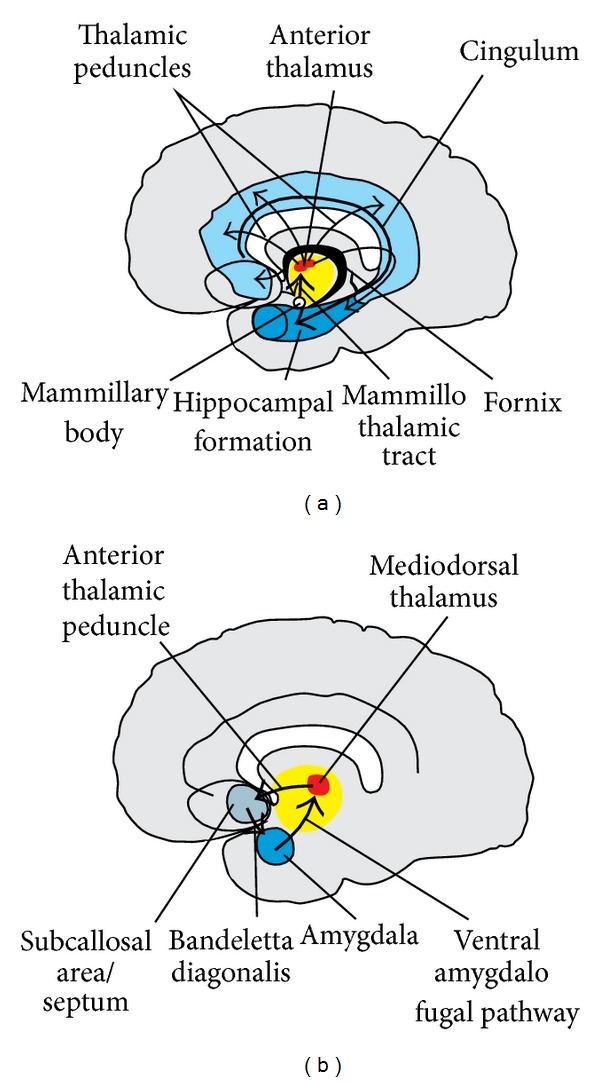
Schematic sagittal sections through the human brain demonstrating the arrangement of the two main circuits, involved in the stages of memory binding and transfer of information for long-term storage. The medial or Papez circuit is shown on the left, and the basolateral limbic circuit on the right. The medial circuit is probably more relevant for the cognitive acts of memory processing, the basolateral one for the affective evaluation of information. Both circuits interact with each other. The Papez circuit interconnects the hippocampal formation via the (postcommissural) fornix to the mammillary bodies, these via the mammillothalamic tract (or tractus Vicq d'Azyr) to the anterior thalamus. The anterior thalamus with its cortical projection targets reaches the cingulate gyrus, and the subicular part of the hippocampal formation and the cingulum fibers in addition project back from the cingulate gyrus into the hippocampal formation. (The precommissural fornix in addition provides a bidirectional connection between the hippocampal formation and the basal forebrain.) The basolateral limbic circuit links the amygdala, mediodorsal thalamic nucleus, and area subcallosa with each other by distinct fiber projections, namely, the ventral amygdalofugal pathway, the inferior thalamic peduncle, and the bandeletta diagonalis.

Indeed, numerous evidence from the last decades confirms the importance of structures such as the anterior and mediodorsal thalamus and the mammillary bodies [262–273], diencephalic fiber tracts [274, 275], the fornix [276–279], the (posterior) cingulate/retrosplenial cortex [280], the amygdala [18, 281–289], and the basal forebrain/septal nuclei [290–292] (aside from the hippocampal formation and surrounding medial temporal cortex mentioned already above). After Papez [259] Livingston and Escobar [293] suggested another circuit within these structures—the basolateral limbic circuit. Cum grano salis, it can be stated that the Papez circuit is more important for fact-based information and the basolateral limbic circuit for affect-based information. Both interact and are directly interconnected (Figure 9).

#### 6.1.1. Diencephalon

As brain lesions are rarely confined to one nucleus, and as the limbic structures are especially heavy interconnected [294], structures of both circuits are usually damaged. Korsakoff's amnesia usually affects medial thalamic structures and the mammillary bodies within the hypothalamus [295]. Bonhoeffer already in 1901 [197] characterized patients with Korsakoff's syndrome as having major anterograde amnesia, retrograde memory defects, disorientation, and confabulatory tendencies. These cardinal symptoms (as Bonhoeffer termed them) of the usually alcohol-abuse-related disease are still valid today. Impairments in consuming and metabolizing vitamin B1 apparently lead to the characteristic brain degenerations in the diencephalon. Intelligence can be largely preserved in Korsakoff's amnesics.

Also in other diencephalic etiologies as in infarct patients, intelligence is unaffected after bilateral damage to structures such as the mediodorsal nucleus, the mammillothalamic tract and the medial internal lamina (containing thalamocortical fibers [27]. Markowitsch et al. [27] even emphasized in the title of their publication that the patient had “preserved intelligence and severe amnesic disturbances.” Furthermore, he usually was unconscious of his amnesia and tended to confabulate. In this way this diencephalically damaged patient differed from the medial temporal lobe-damaged patient H.M. [189, 202, 203, 296] of whom the following statement was passed on: “every day is alone, whatever enjoyment I've had, and whatever sorrow I've had” [296, page 217]. The impairment of our diencephalic patient with respect to conscious self-reflection is in accordance with Dercum's [297] hypothesis that the thalamus is necessary for consciousness, proposed nearly ninety years ago. Dercum [298] attributed consciousness to the fibers ascending from the thalamus to the cortex (page 293). As the thalamus would be the seat of all sensations, perceptions, feelings, and emotions, its activity is necessary for safety, success, and well-being of the organism (page 294). This idea was later confirmed by the findings of other authors [297]. Recently, Philippi and coworkers [59] did an extensive case analysis of a patient with severe nonthalamic damage after a severe episode of herpes simplex encephalitis; they found that while his autobiographical or extended self was at least partially affected by the extensive bilateral damage of insula, anterior cingulate, and medial prefrontal cortices, his core self-awareness (including self-recognition and the sense of self-agency) appeared preserved.

#### 6.1.2. Amygdala

Within the basolateral limbic circuit (cf. Figure 9) the amygdala or amygdaloid complex constitutes a major hub for processing emotional material and providing connotations to new incoming material. In fact the amygdala receives preprocessed information from all sensory modalities and analyzes the social and biological significance for the self [18]. Amygdala damage usually occurs in relation to encephalitis and is nonselective in this condition. There exists, however, a rare disease condition which leads to selective bilateral degeneration (calcification) of the two amygdalae, the Urbach-Wiethe disease. Urbach-Wiethe disease primarily is a dermatological disease condition which, however, in most cases leads in adulthood to amygdala calcification. In the United States of America, there is especially the patient SM who has been studied extensively with respect to deficits resulting from this disease [299–306]. In Europe and South Africa larger numbers of patients with Urbach-Wiethe disease were studied in recent years [18, 20, 282, 285–287, 307, 308].

Common to these reports is the fact that patients with selective bilateral amygdala damage show alterations in identifying what is usually of social and emotional significance, though there is a wide range of interindividual variation [285–287, 307–310]. Also specific form of social—such as altruistic—behavior is altered [309]. The patients appear less sure about their judgments, have olfactory disturbances, and appear with insufficient distance when approaching others [18, 20]. Fear is reduced in them [283]. Interestingly, memory is affected both during learning and recall [285–287, 311, 312] as the patients have difficulty in distinguishing relevant from irrelevant information. Consequently, the amygdala has an important impact on memory processing, especially on episodic autobiographical memory [311–317]. Mayford et al. [316] exemplified this for fear memory and synaptic changes in the amygdala and we [317] have argued “that the amygdala is not simply an emotional brain structure but integrates emotion with cognition” (page 166). As shown in Figure 1 of Markowitsch and Staniloiu [317], we have a somewhat similar approach as Quian Quiroga (cf.  Figure 8) by assuming that networks of single neurons, engaged in different aspects of information processing (such as in arousal and activation, representation, and affect coding), are necessary for successful self-reflected information retrieval. With the example of 9/11, Sharot et al. [318] have demonstrated with functional brain imaging that the amygdala enhances personal-relevance-related activity during the recall of a frightening event.

### 6.2. Information Storage and the Cerebral Cortex

As Figure 7 indicates, the cerebral cortex is considered to be the main storage area for mnestic information. This hypothesis comes from calculation models of its neuronal capacity [319, 320], from evidence from patients with large-scale [321, 322] degeneration of cortical structures [323, 324], and from models of information storage in neural structures [185–187]. Furthermore, lesion research in patients provides some evidence for modularity of memory storage in cortical regions [325–328]. Especially, however, two streams of research provide evidence for widespread cortical storage: for one, evidence from patients with conditions after severe hypoxia [323] and dementia-related cortical degeneration [324] with severe anterograde and retrograde amnesia reinforces the view that the cerebral cortex is the principal storage place for consciously processed information. Secondly, the idea that the synchrony of cortical oscillations serves as a binding phenomenon for memory [329–332]. This synchrony might be promoted by thalamocortical interaction [333].

### 6.3. Information Retrieval and the Temporo-Frontal Cortex

When it comes to the retrieval of consciously processed information, we in 1993 studied a patient with traumatic brain injury of mainly the right temporo-frontal cortex with some less severe contrecoup damage to the respective region of the left hemisphere [334]. The patient had complete retrograde amnesia in the episodic autobiographical domain. However, he was able to learn new information in long-term and he had partly preserved semantic memory, together with fully preserved skills (procedural memory). A few years later we and others found several patients with similar brain damage and a similar inability to retrieve personal events from the past [160, 161, 335, 336]. Vice versa, others and we observed that patients with mainly left hemispheric damage to the same regional combination—which is interconnected bidirectionally by the uncinate fascicle, a fiber tract which seems to grow or increase in size beyond the age of 30 years [337]—had an inability to retrieve past semantic memories [163, 338]. The patient of De Renzi et al. [338] was a 44-year-old school teacher with herpes encephalitis affecting her left anteromedial inferior temporal lobe. She had preserved autobiographical memories and a preservation of grammar and syntax rules but seemed to have forgotten all her world knowledge as tested, for example, with famous names (e.g., Moro, Mussolini, Stalin, Kaddafyi, Hitler, Mozart, Wagner, Garibaldi, Greta Garbo, and Dante). Also recipes for cooking (e.g., spaghetti) had been lost. 

We confirmed the importance of the right temporo-frontal cortex for retrieval with functional brain imaging (positron emission tomography) by studying autobiographical memory retrieval of young normal participants [339]. Again, we observed that retrieval of these events activated largely the right hemispheric temporo-frontal cortex. Our findings were taken up in a review on “Cognitive neuroscience of emotional memory” by LaBar and Cabeza [162] who wrote on page 59 “studies of retrograde amnesia support Markowitsch's proposal that retrieval of remote personal memories involves interactions between the inferior PFC (prefrontal cortex) and its connections with the anteromedial temporal lobe that course through the uncinate fasciculus.” It is at present undecided whether the damage of the temporo-frontal region indeed causes retrograde amnesia in the way that there is no longer access to the probably more posteriorly in the temporal lobe and other cortical-areas-situated old memories, or whether the damage (which frequently occurs via traumatic brain injury) triggers a psychogenic reaction that disrupts conscious access to old memories [cf. [340, 341]]. If the second mechanism were correct, it would still be difficult to interpret the selective semantic amnesia in cases with left hemispheric damage. The right hemisphere has been considered to be more intimately involved in emotional processing [342] which would speak in favor of the episodic autobiographical amnesia after its damage. Of importance is that the self of the patient is considerably disturbed as he or she no longer has access to his or her personal past or to significant portions of it (at least in cases with right hemispheric damage). The same phenomenon occurs in patients with dissociative or psychogenic amnesia and will be discussed in the next section.

## 7. Memory Distortions and Impairments in Psychiatry

That memory disturbances can occur without overt brain damage has been known already in the 19th century (and very likely before) [178]. Goldsmith et al. [343] pointed out that Pliny the Elder (23–79 A.D.) already had talked about “fright” as being one of the causes of partial or total memory loss. The end of the 19th century brought a major impetus in diagnosing patients as having “hysteria” [344–351] and a new wave in diagnosing this condition came with the First World War [352]. Nowadays we have various overlapping terms to describe the symptomatology of amnesic conditions without concomitant brain damage: “psychogenic amnesia” [353], “dissociative amnesia” [354], “functional amnesia” [95, 355–358], and “mnestic block syndrome” [164–169] are expressions used partly interchangeably, though there are of course differences between them. “Functional amnesia” implies that the amnesia has a function for the affected—it is a more neutral term and can include patients with and without additional (manifest) brain damage. “Dissociative amnesia” is the term used in DSM IV-TR [359] to describe a syndrome where the patient is unable to recall important personal information, usually of a traumatic or stressful nature. The amnesia goes beyond normal forgetfulness but cannot be related to brain damage or the effects of drug consumption. The symptoms cause significant distress or impairment in occupational, social, or other important areas of functioning. “Psychogenic amnesia” is largely overlapping with “dissociative amnesia”; it also emphasizes the psychic and stress-related nature of the disease as well as the occurrence of retrograde amnesia. The term “mnestic block syndrome” emphasizes that the memory loss may be reversible and that just the conscious access to them may be blocked. Even anterograde amnesia of a psychic origin was reported [360, 361]. Long-term followups of such persons are rarely published; probably the 19-year followup we did with a former law student who is anterogradely amnesic since 1994 is the longest [360]. It is known that patients with long-term amnesia deteriorate also intellectually, as they have reduced intellectual stimulation and do not memorize new information properly. It is therefore of interest to do long-term followups with both retrogradely and anterogradely amnesic patients with a psychogenic background in order to find out whether they in any way might be more prone to developing dementia than subjects with normal memory conditions [362]. 

In the last years we investigated a considerable number of patients with these and closely related forms of amnesia [120, 165, 167, 356, 363–369]. For all cases we performed an intensive neuropsychological investigation and usually we also did static and functional neuroimaging with them. Fluoro-deoxy-D-glucose positron emission tomographic (PET) investigations were done to obtain evidence for possible metabolic changes in their cerebrum; water PET was used to determine brain changes in relation to memory. By combining the PET results from 14 of such patients [370] we found that in principal the same brain region—namely, the temporo-frontal cortex of the right hemisphere—was reduced in glucose metabolism which was highly active when normal subjects recalled their autobiographical memories [339]. This finding suggests that if it would be possible to stimulate the right temporo-frontal cortex either via psychotherapy or via transcranial magnetic stimulation (or even by deep brain stimulation), personal memories might be reinstated. 

Interesting features of our patients were–aside from their memory blockade as follows:

- an increased susceptibility and personal lability,
- “belle indifference” and lack of effort,
- identity loss and character changes.

Most of the patients already most likely prior to the onset of the dissociative amnesic condition were particularly susceptible to arguments and opinions of others and were easily directed to change their habits. Patient NN of Mar-ko-witsch et al. [120] followed the suggestion of a person from the Salvation Army to enter a psychiatric hospital. He also followed the suggestion of his friend to take her last name also as his new last name after marriage. NN also did not object to his wife to take vacation with the family in spite of the lack of money. He also immediately followed the advice of homeless people to enter the main station of a large city. Furthermore, he was influenced by another patient during his psychiatric stay to change his profession and try to become owner (together with the other patient) of a restaurant. The patient described by Markowitsch et al. [165] throughout his life appeared to act only after the will of his mother and, later, of his wife. The mother suggested to him what he should study, the wife pushed him early in his career to establish an own firm and to buy a house.

The majority of our patients were principally indifferent towards their disease. Most of them refused therapeutic interventions and appeared even satisfied with their new life situation. The patient described in Markowitsch et al. [360] lives since onset of her amnesia in 1994 in a small apartment together with her old mother without trying to leave the house or to meet other people. She just stays with her computer and walks from time to time through portions of the nearby park. We had noted a similar condition with significant and lasting lack of effort with respect to the own condition in several other patients, including a former medical doctor (who developed a compulsive hoarding syndrome), who remained in their life condition in spite of numerous attempts from the environment to change their situation. This symptomatology was also found in a patient without identity loss [371].

Identity loss is the most significant and most frequent concomitant of dissociative amnesic states. The patient no longer knows who she or he was, who his or her partner and children are and has no clue about his or her past emotional behavior (e.g., with respect to intimacy with the partner). Patients, for example, also describe that they do not any more recognize her face in a mirror [120, 368] (a finding described since long) [372]. Patient NN of Markowitsch et al. [120] changed his life habits: before his amnesia he was an avid car driver, afterwards he considered cars as too fast for human beings and avoided sitting in them. He also changed his diet and eating habits.

Also direct somatic changes were observed: patient NN [120] after amnesia onset lost his asthma and allergy. Another patient, who had been accused of raping a female, lost his ability to urinate and had to be catheterized by his wife during day and night time [373].

Interestingly, many of such changes, affecting self-consciousness and self-awareness, were already noted in the frequently painstakingly careful descriptions of the old days (e.g., [345, 346, 348, 374–376]), though sometimes different expressions were used. Interesting is, for instance the frequently used term of “double consciousness” [377–380], which partly reflects the general dissociation in mental state and partly the possible occurrence of dissociative identity disorders. Even in the belletristic literature of that time (or half a century earlier) reference was made to phenomena resembling dissociative states and depersonalization [381, 382].

Already in 1906 Gordon [383] stated that “self-consciousness is a *conditio sine qua non* of normal life” (page 480) and that amnesia is the most typical of all disturbances of consciousness. Both cases in the old literature as well as some of our recently studied patients show very similar changes after onset of their “mnestic block syndrome”. Dana [379] described in 1874 a 24-year-old “active, intelligent, and healthy… man” (page 571). Due to carbon monoxide poisoning by domestic gas, his behavior changed. After been rescued, he was quite disturbed and “did not know who he was or where he was, and… his conscious memory for everything connected with his past life was gone” (page 572). His behavioral changes included the use and understanding of only the simplest words. After realizing where he was he “pronounced many of the new words with a German accent” (page 572), similar to the way his German attendant spoke. He did not know what marriage meant and did not remember (previously) familiar persons though he seemed especially glad to see them. At first he was unable to read but soon learned to read and write simple sentences. Old musical memories existed and so did habits connected with courtesy.

Interestingly, “in argument he showed considerable dialectic skill and logical power, [but] he evidently could not understand any conceptions at all abstract” (page 573). “The moon, the stars, the animals, his friends, all were mysteries he impatiently hastened to solve” (page 574). While at the beginning he did not recognize his parents or sisters, or his fiancée, from the beginning he said that he had always known her and subsequently his thoughts and feelings centered on her.

Exactly three months after his attack, his memory was completely restored. The recovery started during a visit in the evening at his fiancée's; she thought he was in a condition worse than during the previous months and said “he felt as though one half of his head was prickling and numb, then the whole head, then he felt sleepy and was very quiet… At about 11 o'clock he woke up and found his memory restored… He knew all his family at once and was plainly just the same man as before. But the three months was an entire blank to him.” (page 575). So he did not know Professor Dana whom he had not seen before the accident.

While such an abrupt and nearly complete memory restoration, the total but time-limited loss of knowledge about his family and friends, and the age of the patient all favor psychogenic amnesia, the carbon monoxide poisoning provides a basis for an organic damage-based explanation, possibly the poisoning, but triggered the release or manifestation of an already existing tendency for psychogenic memory loss. 

Our recently investigated patient with complete retrograde amnesia in the episodic autobiographical domain and an additional brain glioma was a 29-year-old male [369]. He had a several-week history of gait disturbances, headaches, and intermittent problems with his vision. A medulloblastoma occupying the fourth ventricle was diagnosed and subsequently completely removed surgically. Postoperatively the patient did not recognize his friends, his grandmother, and even himself in pictures from his youth. He only recognized his parents and his brother. He forgot the profession of his father. He did not know the names of his acquaintances, friends, or former colleagues anymore. He did not know which professional training or job he had had. He did not recall that fishing used to be his hobby. Furthermore he did not know how to use a fishing rod. He encountered considerable difficulties with naming objects. He perceived his situation as if he stood in a white room, where the door behind him had been locked and he did not know where he had come from. Tests of anterograde memory were in the normal range or even above average. His past history revealed that he had been adopted after birth and had received treatment in childhood for difficulties with concentration and a delay in language development. At the age of 26 years the patient was diagnosed with a testicular carcinoma, which was completely surgically removed, and also treated with both chemotherapy and radiation therapy; he had had several other diseases of the scrotum when he was a child. 

Similarly to Dana's patient this case is a prototypical illustration of the complex interplay between organic and psychogenic mechanisms that underlies several cases of retrograde amnesia. These cases have often been listed under the diagnostic construct of functional amnesia.

Functional amnesias demonstrate to what degree the environment can shape the nervous system. An adverse environment can alter the brain's metabolism and—in the longer run—lead to structural changes. The science of epigenetics provides a convincing basis for such an interplay [73–88]. An example from neurology is transient global amnesia, a condition of sudden memory loss.

## 8. Brain, Memory, and Self

Both the case descriptions of patients with an organic and with a psychogenic basis of their memory loss reveal that memory is an essential attribute of our well-being and our personality. Memory allows capturing the world in space and time and to mentally travel into various epochs of one's past life. In addition it allows “proscopic chronesthesia”—the forward-looking sense of subjective time [384]. Anterograde amnesia leaves the patient “frozen in time”—he or she can no longer acquire emotionally colored information which would allow adapting towards new circumstances. Retrograde amnesia on the other hand leaves a person without grounding, without orientation on his or her background. There is no reference available for being similar to or different from others. 

Patients with memory loss have deficits with respect to their possibilities of self-reflection and personal temporality [385–387]; other attributes such as self-agency and self-ownership can be seen as largely preserved. Klein [388] recently argued that there are several episodic memory enabling systems—ownership, self, subjective temporality, and agency—which are necessary for episodic autobiographical recollection. Vice versa, one can argue that an impairment in episodic autobiographical recollection, whether due to overt damage of neural tissue or whether due to stress-related glucocorticoid (stress hormone) release, can retroactively affect the self.

Self and episodic autobiographical are dynamic processes, shaped over the life time by the ups and downs of brain development (myelination, pruning, sexual dimorphism, neurogenesis, synaptogenesis, vessel growth, and regression) [389–398], the social-cultural influences on brain and self over time [399–402], and especially during aging [403–405]. As our brain differs across life time, our self changes as well. We may not (always) be aware of these changes, as we cannot monitor our self from an external point of view. Nevertheless, we can observe and test such changes by viewing others. And above all, brain damage—whether focal [19–23, 26, 27, 406] or extensive (degenerative or diffuse) [407–410]—makes such changes visible even to the layperson.

There is evidence from comparative biological [5], clinical neurological [59], and memory research [2, 4] that the complex interplay of self and brain can be altered at many levels (including drug application [411, 412]). We still have only begun to unravel the brain processes responsible for memory consolidation [413–416], storage, and retrieval [242, 417, 418]. All the more we are only starting to disclose relations between brain and mind [3, 6, 419–421].

## Figures and Tables

**Table 1:** Psychological processes associated with memory.

	Description of memory process
Registration	The conscious or unconscious perception of stimuli via the sensory organs

Encoding	The initial processing and transfer of perceived information along the relevant sensory relays (nuclei, areae) of the nervous system; short-term storage (4–7 bits or seconds to minutes)

Encoding specificity principle	Assumption that specific information (item) is encoded in a specific environmental context and therefore this item can be best retrieved when the same or similar context is present (state-dependent encoding and retrieval of information)

Consolidation	The processes that bind, associate, and restructure new material for long-term storage (might be aided by sleep)

Storage	The main embedding of new material in the neuronal network (so-called engrams; thought to be a result of protein synthesis and morphological changes)

Retrieval	The activation and recovery of stored material, also termed ecphory;* environmental stimuli or thought processes trigger the process of retrieval/ecphory
Free recall	The volitional generation of information that occurs without external help, cueing, or aiding (e.g., unaided recall of who the prime minister of the UK is)
Cued recall	Recall that occurs with the help of external stimuli (e.g., recall of a name when told the first letter)
Recognition	Correct identification (or selection) of a stimulus when the intact stimulus is presented again together with several other stimuli (choices) that have features or attributes in common with the original (e.g., selection of the correct photograph when shown several similar pictures)
Primacy effect	The finding that the first items on a long list of encoded items are disproportionally well recalled, indicating that they were already transferred into long-term memory
Recency effect	The finding that the last items on a long list of encoded items are disproportionally well recalled, indicating their sustained rehearsal in short-term memory, an indicator of short-term memory performance.

Postretrieval reencoding	(Conscious) information retrieval leads to immediate reencoding on the basis of present internal and external circumstances (possibly implying a subsequent changed representation)

Postretrieval reconsolidation	After retrieval, memories are assumed to enter a transient labile phase followed by a process of stabilization

*Tulving [67, 68] used the term ecphory to describe the process by which retrieval cues interact with stored information so that an image or a representation of the information in question appears. Ecphory implies a state-dependent retrieval of information (and is consequently affected by mood as well).
